# Distribution of neurotological findings in patients with cochleovestibular dysfunction

**DOI:** 10.1016/S1808-8694(15)31325-2

**Published:** 2015-10-20

**Authors:** Edmir Américo Lourenço, Karen de Carvalho Lopes, Álvaro Pontes, Marcelo Henrique de Oliveira, Adriana Umemura, Ana Laura Vargas

**Affiliations:** ^1^ Joint Professor, Ph.D., Responsible for the Discipline of Otorhinolaryngology, Medical School, Jundiaí; ^2^ Resident Physician, Discipline of Otorhinolaryngology, Medical School, Jundiaí; ^3^ Resident Physician, Discipline of Otorhinolaryngology, Medical School, Jundiaí; ^4^ Resident Physician, Discipline of Otorhinolaryngology, Medical School, Jundiaí; ^5^ Resident Physician, Discipline of Otorhinolaryngology, Medical School, Jundiaí; ^6^ Resident Physician, Discipline of Otorhinolaryngology, Medical School, Jundiaí

**Keywords:** otoneurology, vectoelectronystagmography, syndromic diagnosis, labyrinthopathies

## Abstract

**T**he relationship between spatial body positioning and environment comes from perfect corporal balance. The three most important systems responsible for this relationship are: the optic system (sight), the proprioceptive system, and the labyrinthine system. **Study design**: retrospective clinical. We carried out a retrospective study in 3,701 patients of a private otolaryngologic clinic in Jundiai – Sao Paulo, Brazil, who underwent vestibular and cochlear labyrinthine function testing, from 1979 to 2004. **Aim**: To determinate the syndromic distribution of the population and to correlate its relationship with sex, age, symptomatology, as well as otological, clinical and electronystagmographic findings, and which were the most frequent medical specialties who asked for this investigation. **Results**: We found higher prevalence in females (1.75:1). Seventy-nine percent of the patients were aged 20 to 59 years old, therefore including people in productive age, with a major prevalence of peripheral syndromes, but there was no preference for age or sex among different syndromes. This study also demonstrated that some otoneurological symptoms were common to all kinds of otoneurological syndromes, in opposition to the data found in the world literature. Tinnitus, hearing loss, nausea and vomiting as well as harmonic alterations in clinical examination were found with more frequency on peripheral syndromes, whereas non-harmonic was found in central syndromes, according to the reviewed literature. The conclusions showed that the majority of the patients started their investigation with either otolaryngologists or neurologists and 36% of the patients had peripheral syndrome and almost 25% had normal evaluation.

## INTRODUCTION

Balance, from Latin *aequilibriu*, means maintenance of the body in a normal posture or position without oscillation or deviation. Body balance is essential for spatial relation of the body in the environment. Balance disorders make the patients unsure and produce severe quality of life affections. Three systems are responsible for maintaining balance: vision, proprioceptive system and vestibular system. It comprises the labyrinth, vestibular nuclei and pathways, which are interrelated at the brainstem region with other neuronal nuclei and pathways, including the cerebellum^1^. Dizziness may arise from different causes, and many functional diseases or disorders from different parts of the body may affect body balance system. No isolated clinical signal have a definite value in localizing the lesion^2^.

Vestibular examination analyzes the functioning of the labyrinth and its correlations with other organs and systems, making it a fundamental part of the otoneurological assessment^3^. The preparation of an accurate anamnesis allows the definition of the syndromic diagnosis and sometimes even the etiological diagnosis of otoneurological clinical presentations^4^. Approximately 85% of dizziness cases are caused by vestibular system dysfunction, either peripheral or central, but dizziness from peripheral vestibular pathologies may be similar to dizziness from central vestibular pathology^5^. Neurovegetative symptoms, such as nausea, vomiting, cold sweating and sometimes diarrhea, are reported by many patients with otoneurological symptoms4., 6., 7., with low incidence in central vestibular syndromes^2^. Central syndromes may manifest or not neurological symptoms and signs^5^.

Complete otoneurological exam includes anamnesis, ENT and neurological physical examination, pure tone audiometry, immittanciometry when necessary, otoneurological tests with vectoelectronystagmography. Predefined response patterns allow the formulation of a syndromic diagnosis, that is, the topographic diagnosis.

Therapeutic management, be it diet, clinical, surgical approach or vestibular rehabilitation depends on the information collected from a detailed anamnesis, combined with information collected from the vestibular exams. Specific and careful diagnosis is the key for successful management^5^.

The purpose of the present study was to conduct a retrospective study of a large sample to define the otoneurological syndromic distribution and to correlate it with gender, age range, anamnesis data, physical examination and multiple findings of otoneurological tests with vectoelectronystagmography, in addition to listing the medical specialties that most frequently referred the patients for specific balance assessment.

## MATERIAL AND METHOD

We analyzed the medical charts of 3,701 patients that underwent detailed anamnesis and clinical examination, followed by cochleovestibular recording using vectoelectronystagmography in a private practice in Jundiaí, state of Sao Paulo, between 1979 and 2004 (25 years).

We distributed patients by age ranges, as follows: from 2 years to 9 years and 11 months (childhood), from 10 to 19 years and 11 months (adolescence), from 20 to 39 years and 11 months (young adults), from 40 to 59 years and 11 months (adults), and over 60 years (elderly).

Medical charts were individually assessed to include data in a Table with many different columns, including gender, age, profession, presence or not of dizziness, tinnitus and neurovegetative signs, affected or normal hearing, affections in part of the tests, syndromic diagnosis (normal, peripheral, central, mixed and uncharacteristic) and medical specialty of the physician who required the assessment. Medical charts with incomplete data were excluded. Hearing analysis was based on pure tone audiometry that was routinely attached to the chart.

Complete otoneurological exam comprised: 1) anamnesis; 2) ENT and neurological physical examination using segmental tests such as Romberg, Unterberger (static gait) and Indication (stretched arm test), dysmetria, diadochokinesia and cranial nerves, observation of spontaneous and semi-spontaneous nystagmus (directional); 3) pure tone audiometry, immittanciometry, if necessary, to complement data; 4) otoneurological data with vectoelectronystagmographic records including spontaneous, semi-spontaneous, positional, optokinetic, horizontal and vertical nystagmus, pendular and circular tracking, decreasing pendulum rotary chair tests and caloric evaluation with air stimuli at 20º C and 42ºC. Response patterns were predefined and served as reference for the conclusions, allowing the formulation of topographic diagnosis.

Based on the found results, we reached a syndromic distribution of all the studied population, which was compared to other data collected in the study. Prevalence and cross matching of data was carried out with a database created in the software Access Office 2000. Data were processed in this software and final results were exported to software Excel Office 2000, used to compile Charts and Tables.

## RESULTS

Out of the total number of patients, 2,353 were female and 1,348 were male patients, as shown in Table 2 and Charts 2A, 2B, 2C and 2D.

**Table 2:** Syndromic and gender distribution of patients submitted to complete otoneurological assessment.

Syndromic diagnosis	FEMALE	MALE	Total
1 – normal	615	349	964
2 – peripheral	845	464	1309
3 – central	447	267	714
4 – mixed	317	182	499
5 – uncharacteristic	129	86	215
Total	2353	1348	3701

**Chart 2A:**
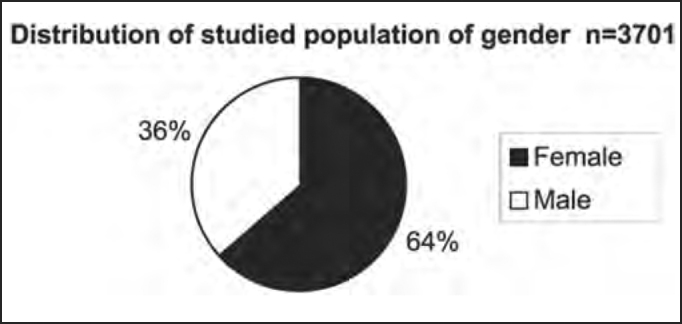
Distribution of studied population according to gender.

**Chart 2B:**
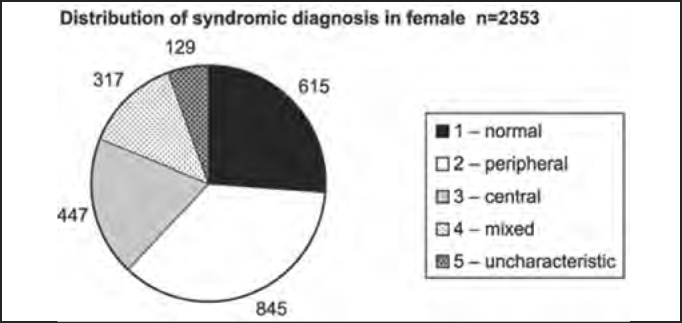
Distribution of syndromic diagnosis in female patients submitted to complete otoneurological assessment.

**Chart 2C:**
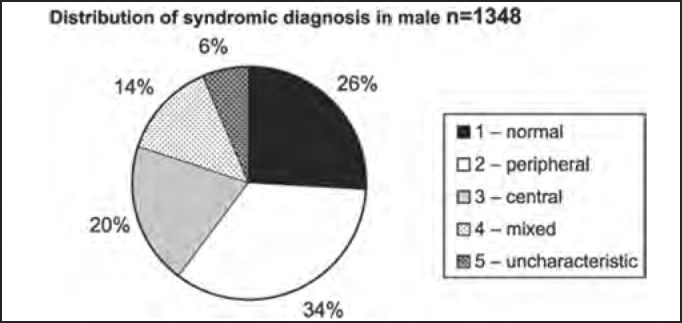
Distribution of syndromic diagnosis in male patients submitted to complete otoneurological assessment.

**Chart 2D:**
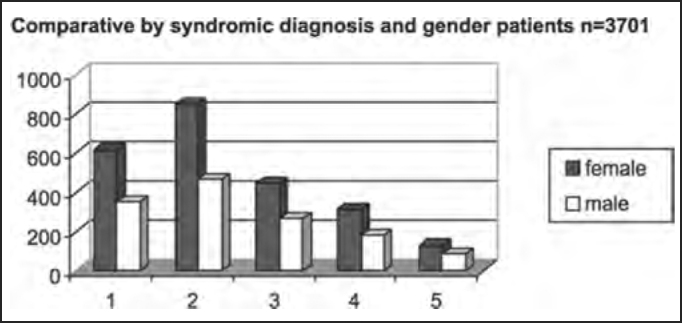
Comparative distribution by syndromic diagnosis and gender in patients submitted to complete otoneurological assessment. 1 – normal; 2 – peripheral; 3 – central; 4 – mixed; 5 – uncharacteristic.

**Chart 3:**
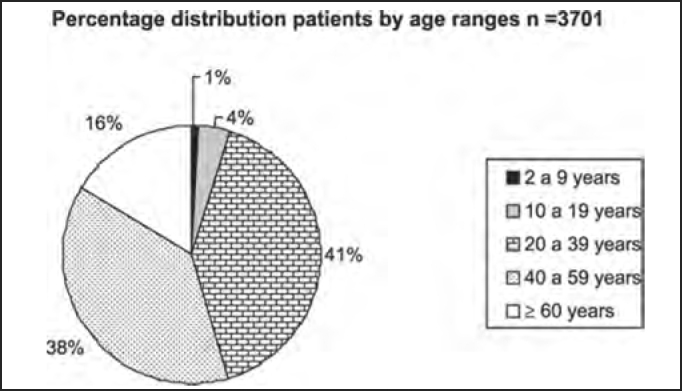
Percentage distribution by age ranges (in years) of 3,701 patients submitted to complete otoneurological assessment.

The percentage distribution of the studied population in age ranges and syndromic diagnosis is shown in Table 3 and Chart 3.

**Table 3:** Numeric and percentage distribution by age ranges of 3,701 patients submitted to complete otoneurological assessment.

Age range	2 to 9 years	10 to 19 years	20 to 39 years	40 to 59 years	³ 60 years	Total
n	27	143	1514	1408	609	3701
estimated %	1	4	41	38	16	100

The distribution by syndromic diagnosis and age ranges (in years) of the studied population in shown in Table 4 and Charts 4A, 4B, 4C, 4D, 4E and 4F.

**Table 4:** Distribution by syndromic diagnosis and age range (in years) of patients submitted to complete otoneurological assessment

SYNDROME	2 to 9	10 to 19	20 to 39	40 to 59	≥ 60	Total
1 – normal	15	61	481	319	88	964
2 – peripheral	6	36	518	538	211	1309
3 – central	3	29	261	270	151	714
4 – mixed	3	12	154	202	128	499
5 – uncharacteristic	0	5	100	79	31	215
Total	27	143	1514	1408	609	3701

**Chart 4A:**
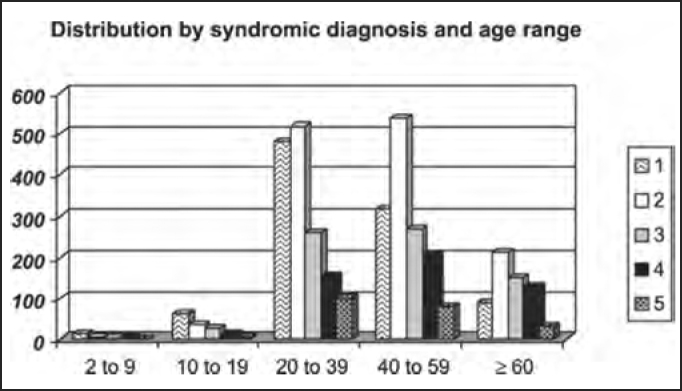
Distribution by syndromic diagnosis and age range (in years) of all patients submitted to complete otoneurological assessment. 1 – normal; 2 – peripheral; 3 – central; 4 – mixed; 5 – uncharacteristic.

**Chart 4B:**
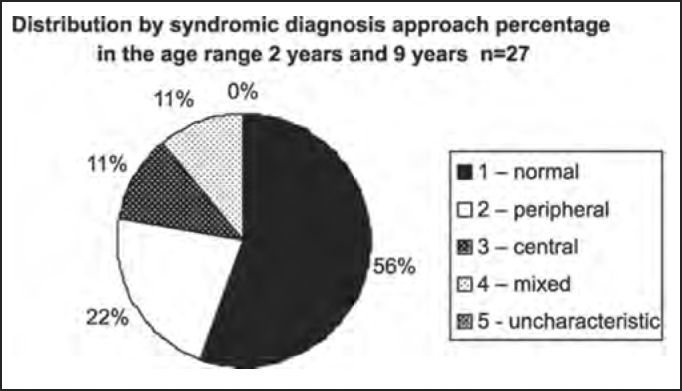
Distribution by syndromic diagnosis of all patients submitted to complete otoneurological assessment in the age range 2 years and 9 years and 11 months.

**Chart 4C:**
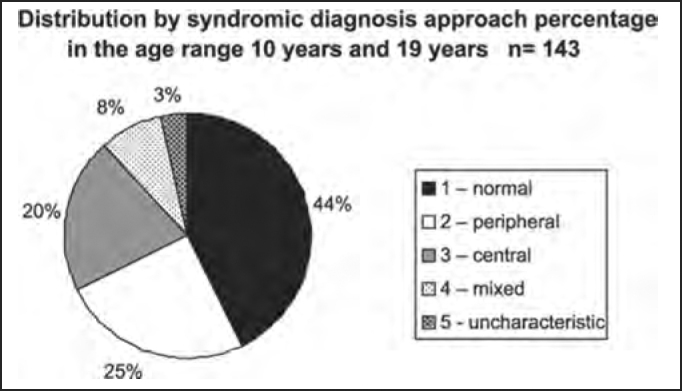
Distribution by syndromic diagnosis of all patients submitted to complete otoneurological assessment in the age range 10 years and 19 years and 11 months.

**Chart 4D:**
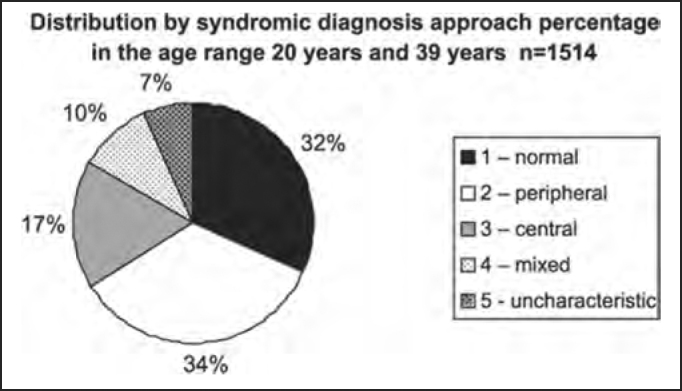
Distribution by syndromic diagnosis of all patients submitted to complete otoneurological assessment in the age range 20 years and 39 years and 11 months.

**Chart 4E:**
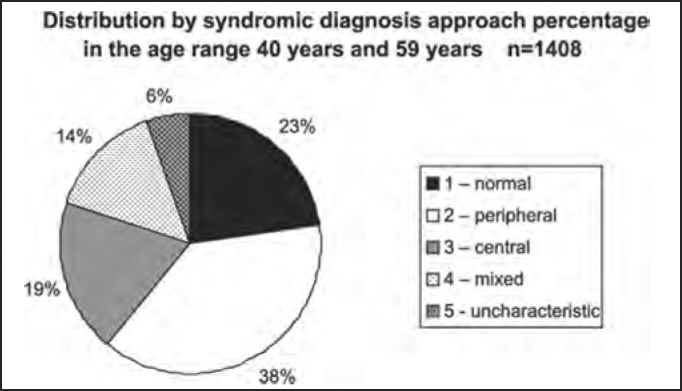
Distribution by syndromic diagnosis of all patients submitted to complete otoneurological assessment in the age range 40 years and 59 years and 11 months.

**Chart 4F:**
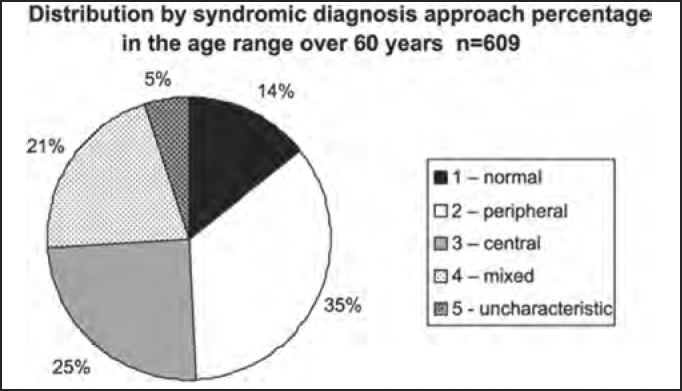
Distribution by syndromic diagnosis of all patients submitted to complete otoneurological assessment in the age range over 60 years.

## DISCUSSION

**Chart 1:**
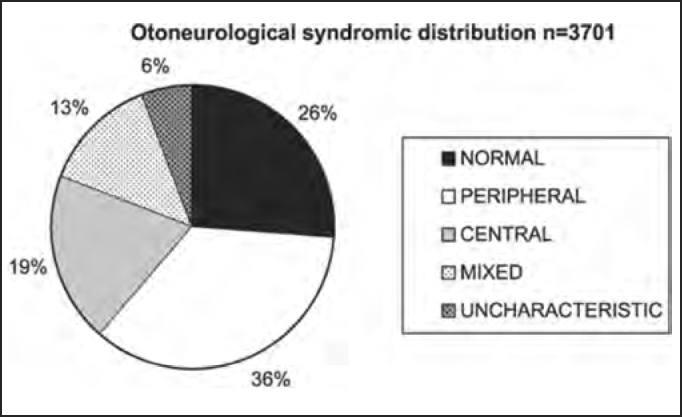
Otoneurological syndromic distribution of the whole studied population and approximate percentages.

Different signs of vestibular dysfunction may be detected with anamnesis associated with clinical examination and complete otoneurological or vestibulometric assessment using vectoelectronystagmography. One or more tests may present affections, allowing the formulation of a diagnostic hypothesis of peripheral vestibular syndrome (uni or bilateral, irritative or deficit), central, mixed or uncharacteristic; nevertheless, many patients did not present any abnormality in the test. Clinically speaking, dizziness in peripheral vestibulopathy may be similar to dizziness in central vestibulopathy. As shown in Table 1 and Chart 1, about 74% of the 3,701 studied patients presented vestibular system dysfunction, be it peripheral, central, mixed or uncharacteristic, a finding that is partially disagreeing in the literature, which reports about 85%^8^. In the assessment of syndromic distribution, we found higher prevalence of peripheral syndrome (36% of the cases), followed by central affection (19%), mixed (13%), and uncharacteristic (6%), as well as 26% of normal exams. These data do not differ from those found in the literature, which defined peripheral system affection as the main cause of otoneurological symptoms2., 7..

**Table 1:** Otoneurological syndromic distribution of the whole studied population and approximate percentages.

Syndrome	n	%
Normal	964	26
Peripheral	1309	36
Central	714	19
Mixed	499	13
Uncharacteristic	215	6
Total	3701	100

Next we show the distribution by syndromic diagnosis of patients with and without dizziness, tinnitus and neurovegetative signs reported in the anamnesis submitted to complete otoneurological assessment.

The presence of auditory affections were confirmed by audiometry, regardless of the report provided by the patients in the anamnesis. The results and findings to the tests of the physical examination are shown next.

Indication of specialized otoneurological assessment requested by different medical specialties can be seen next.

There was predominance of female subjects in the studied population in the proportion of 1.75:1 (Table 2 and Chart 2A), a finding that is in agreement with the massive study of vestibulocochlear diseases^7^. As to syndromic distribution by gender, there was no discrepancy between them, and peripheral syndrome was the most frequent in both genders (Table 2 and Charts 2B, 2C and 2D).

In the analysis of syndromic distribution by age range, it was evident that most of the exams were required for people aged between 20 and 59 years (Table 3 and Chart 3). Differently from what was initially expected, the age range 20 to 39 years included most of the patients with different types of vestibular syndromes, followed by the age range 40 to 59 years and not the elderly. It may have some repercussions in social-economic status, because it tends to temporarily or permanently disables subjects of the population that are at their most productive period in life (Table 4 and Chart 4A, Chart 4B, Chart 4C, Chart 4D, Chart 4F) and this fact may be correlated with the increasingly stressing pace of modern life. It was observed that in childhood and in adolescence exams were predominantly normal, maybe because psychoemotional problems are the most prevalent in this age range. Upon observing the syndromic distribution, it was quite similar in age ranges 20 to 39 and 40 to 59 years, with higher predominance of peripheral syndrome, followed by normal cases. Even patients older than 60 years presented more cases of peripheral syndrome than central affection (Table 4 and Chart 4F).

**Chart 5:**
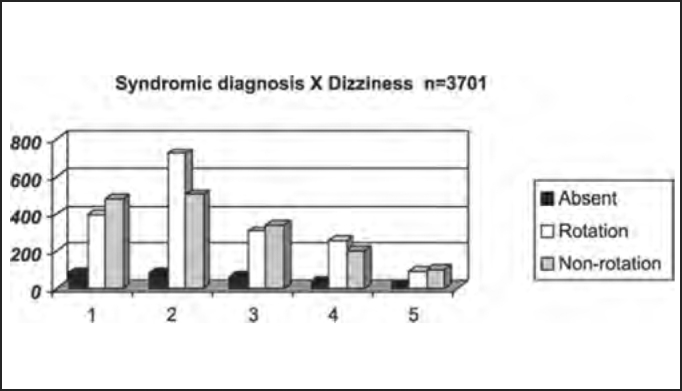
Distribution by syndromic diagnosis of patients with and without rotation dizziness in the anamnesis submitted to complete otoneurological assessment. Syndromic diagnosis: 1 – normal; 2 – peripheral; 3 – central; 4 – mixed; 5 – uncharacteristic.

As to symptomatology of dizziness, tinnitus, neurovegetative symptoms (SNV), hearing disorders and their correlation with syndromic distribution, there was higher frequency of rotation dizziness (721 or 40.6% in 1,776 patients) and also non-rotation dizziness (502 or 30.8% in 1,628 patients) in peripheral syndromes, suggesting that patients that complained of dizziness of any kind had higher likelihood of having peripheral diseases and not central diseases, but we could not correlate type of dizziness with syndromic diagnosis. Only 8% of the studied population (297 patients) did not complain of dizziness (Table 5 and Chart 5).

**Table 5:** Distribution by syndromic diagnosis of patients with and without rotation dizziness in the anamnesis submitted to complete otoneurological assessment.

DIZZINESS	Absent	Rotation	Non-rotation	Total
1 – normal	88	395	481	964
2 – peripheral	86	721	502	1309
3 – central	65	308	341	714
4 – mixed	35	260	204	499
5 – uncharacteristic	23	92	100	215
Total	297	1776	1628	3701

**Chart 6:**
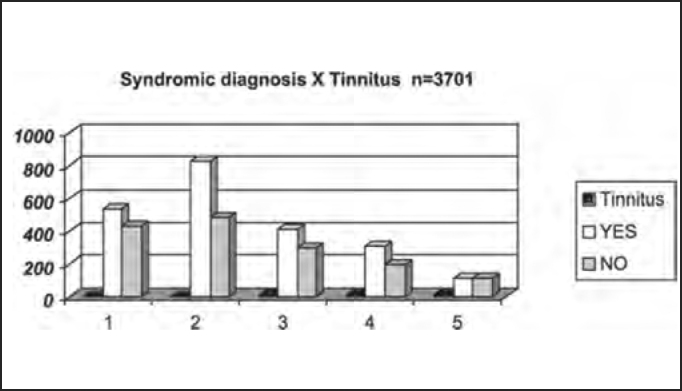
Distribution by syndromic diagnosis of patients with and without tinnitus in the anamnesis submitted to complete otoneurological assessment: 1 – normal; 2 – peripheral; 3 – central; 4 – mixed; 5 – uncharacteristic.

Tinnitus complaint was detected in 59.2% of the studies population, predominantly in patients with peripheral findings, but it was present in 11.2% of the central cases, a finding in agreement with the report by Ganança et al.^8^, who stated that peripheral syndromes normally present sensation of ear pressure or discomfort (Table 6 and Chart 6).

**Table 6:** Distribution by syndromic diagnosis of patients with and without tinnitus in the anamnesis submitted to complete otoneurological assessment.

Tinnitus	YES	NO	Total
1 – normal	535	429	964
2 – peripheral	823	486	1309
3 – central	416	298	714
4 – mixed	309	190	499
5 – uncharacteristic	108	107	215
Total	2191	1510	3701

**Chart 7:**
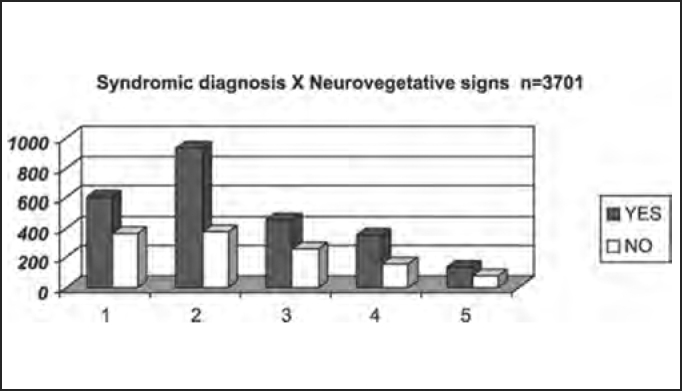
Distribution by syndromic diagnosis of patients with and without neurovegetative signs in the anamnesis submitted to complete otoneurological assessment. Syndromic diagnosis: 1 – normal; 2 – peripheral; 3 – central; 4 – mixed; 5 – uncharacteristic.

There was predominance of SNV (about 2/3 of the studied population), who were most of the cases patients with peripheral affections, a finding that is in agreement with the studied literature^8^, which reported low incidence of the symptom in central vestibular syndromes, but 67% of the population in the study had SNV, divided into 37.9% of peripheral cases and 18.4% of central cases (Table 7 and Chart 7), a finding that is not in agreement with the investigated literature3., 5., 6., 9.. The exception lies with cerebellum-affected patients, because they frequently have response hyperreflexia, associated with SNV.

**Table 7:** Distribution by syndromic diagnosis of patients with and without neurovegetative signs (SNV) in the anamnesis submitted to complete otoneurological assessment.

Neurovegetative Symptoms	YES	NO	Total
1 – normal	599	365	964
2 – peripheral	940	369	1309
3 – central	457	257	714
4 – mixed	346	153	499
5 – uncharacteristic	137	78	215
Total	2479	1222	3701

**Chart 8:**
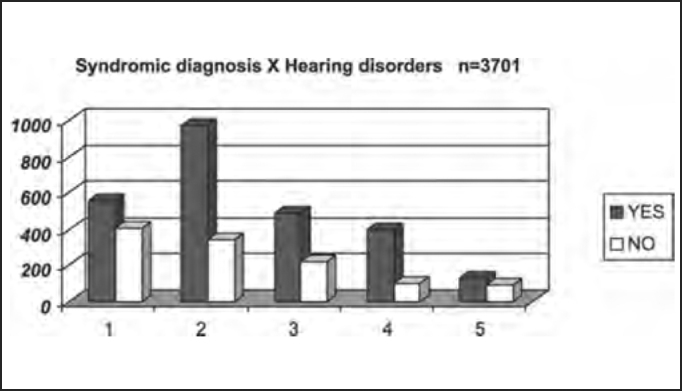
Distribution by syndromic diagnosis of patients with and without hearing disorders in the audiometry submitted to complete otoneurological assessment. Syndromic diagnosis: 1 – normal; 2 – peripheral; 3 – central; 4 – mixed; 5 – uncharacteristic.

The presence of hearing difficulties occurred in 68.7% of the studied population, confirmed by audiometry. Out of the total, 38.1% had peripheral findings, in agreement with Ganança et al.^8^, who stated that peripheral syndromes normally do not present hearing loss and these cases may be added by others that have mixed or uncharacteristic syndromes, that is, no clearly defined topodiagnosis (Table 8 and Chart 8).

**Table 8:** Distribution by syndromic diagnosis of patients with and without hearing disorders in the audiometry submitted to complete otoneurological assessment.

Hearing disorder	YES	NO	Total
1 – normal	559	404	964
2 – peripheral	969	341	1309
3 – central	490	224	714
4 – mixed	399	100	499
5 – uncharacteristic	126	89	215
Total	2543	1158	3701

In summary, as to symptomatology, the study demonstrated the presence of otoneurological symptoms that are common to different types of otoneurological syndromes, such as for example, no classical predominance, as shown in the literature, which indicates rotation dizziness as highly suggestive of peripheral affection and non-rotation as of central involvement; in addition, it confirmed the importance of tinnitus as a symptom, as an indication for otoneurological assessment.

**Chart 9:**
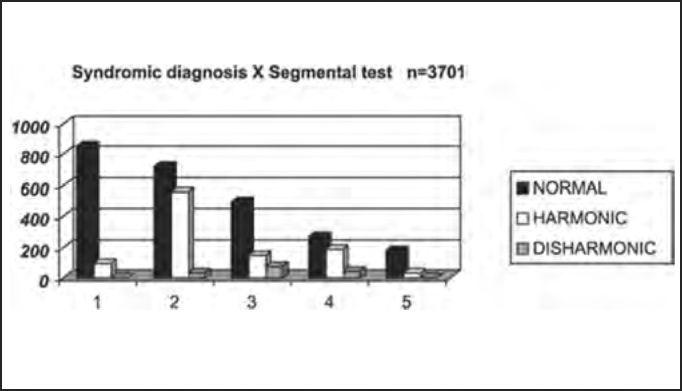
Distribution by syndromic diagnosis of patients with and without abnormal segmental test results in the physical examination submitted to complete otoneurological assessment. Syndromic diagnosis: 1 – normal; 2 – peripheral; 3 – central; 4 – mixed; 5 – uncharacteristic.

As to assessment of the importance of segmental tests in the syndromic diagnosis of the studied population, the study showed the presence of affections, either harmonic or disharmonic, in a total of 32% of the patients. Subjects with central syndrome presented higher prevalence of disharmonic affections to segmental tests and those with peripheral affections showed higher prevalence of harmonic affections to segmental tests; nevertheless, absence of harmonic or disharmonic affections, as detected in 68% of the cases, did not exclude the possibility of the disease. We concluded that segmental tests collaborated for the definition of the syndromic diagnosis in patients with disorders of body balance, and harmonic segmental tests are more frequent in peripheral syndromes, whereas disharmonic segmental tests are more indicative of central syndrome (Table 9 and Chart 9).

**Table 9:** Distribution by syndromic diagnosis of patients with and without abnormal segmental test results in the physical examination submitted to complete otoneurological assessment.

Segmental test	Normal	Harmonic	Disharmonic	Total
1 – normal	855	100	9	964
2 – peripheral	720	553	36	1309
3 – central	493	144	77	714
4 – mixed	269	187	43	499
5 – uncharacteristic	179	34	2	215
Total	2516	1018	167	3701

The authors agree with the statement that no clinical isolated signal has definite value in location of lesion^3^, but they also add that few isolated otoneurological findings can make the topodiagnosis, such as for example drop of one of the upper limps, with absence of orthopedic problems in the indication tests, unilateral dysmetria and unilateral diadochokinesia are highly suggestive of homolateral cerebellum vestibulopathy.

**Chart 10:**
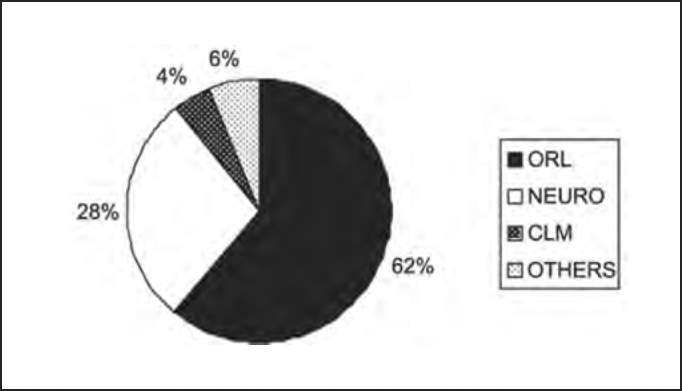
Numeric and estimated percentage distribution of the studied population submitted to complete otoneurological assessment according to specialty of the requesting physician. ORL - Otorhinolaryngology.

**Chart 11:**
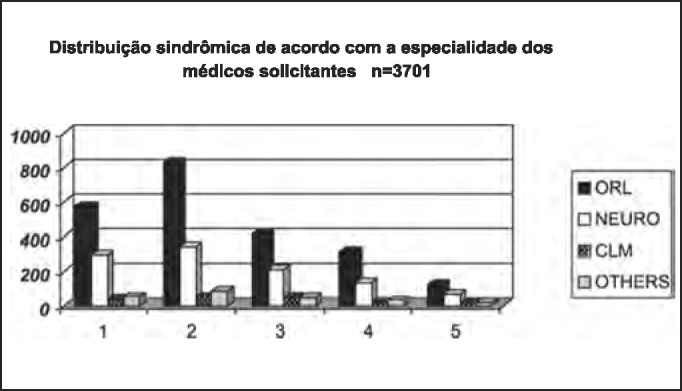
Syndromic distribution of the studied population submitted to complete otoneurological assessment according to specialty of the requesting physician. ORL - Otorhinolaryngology

As to medical specialty of professionals that required otoneurological assessments of the studied population, Table 10 and Chart 10 demonstrated the numeric and percentage distribution, confirming that the Otorhinolaryngologist (ORL) was responsible for 62% of indications in all diagnostic groups, followed by Neurologist with 28%, data that are different from those reported by Sekitani et al. that reported that only 1- to 15% of the patients with dizziness initially looked for an Otorhinolaryngologist^9^. As to syndromic distribution of the population submitted to complete otoneurological assessment according to specialty of requesting physician, we did not observe any tendency of diagnosis of peripheral disease in cases referred by the Otorhinolaryngologist as opposed to central cases referred by the Neurology, as shown in Table 11 and Chart 11.

**Table 10:** Numeric and estimated percentage distribution of the studied population submitted to complete otoneurological assessment according to specialty of the requesting physician.

Medical specialty	n	%
Otorhinolaryngology	2262	62
Neurology	1042	28
General Practitioner	166	4
Others specialties	231	6
TOTAL	3701	100

**Table 11:** Syndromic distribution of the studied population submitted to complete otoneurological assessment according to specialty of the requesting physician.

Syndrome	ORL	NEURO	CLM	Others	Total
1 – Normal	578	295	38	53	964
2 – Peripheral	831	340	53	85	1309
3 – Central	414	210	45	45	714
4 – Mixed	316	132	19	32	499
5 – Uncharacteristic	123	65	11	16	215
Total	2262	1042	166	231	3701

## CONCLUSION

The analysis of 3,701 otoneurological clinical and cochleo-vestibular exams with vectoelectronystagmography led us to the following conclusions:

1. Peripheral vestibular syndrome was the most prevalent one.
2. About ¼ of the patients did not show any vestibular syndromic affection.
3) We detected similar syndromic distribution for both genders, but more prevalent in female subjects at the proportion of 1.75:1.
4. The most frequently affected age range was 20 to 39 years, followed by 40 to 59 years; however, the most prevalent syndrome in both was peripheral syndrome. In childhood and adolescence, most of the exams were normal. The higher prevalence of central syndrome was observed in the age range over 60 years.
5. Complaints of dizziness of any kind showed higher likelihood of peripheral than central affection, but there was correlation between type of dizziness and syndromic diagnosis.
6. Hearing disorders, presence of tinnitus and neurovegetative symptoms showed predominance in peripheral syndromes.
7. There was higher frequency of harmonic deviations in segmental tests in patients with peripheral syndrome than disharmonic deviations in patients with central syndrome.
8. The medical specialty that has more frequently requested vestibular assessment was Otorhinolaryngology, followed by Neurology.
